# How to recognize clustering of luminescent defects in single-wall carbon nanotubes†

**DOI:** 10.1039/d4nh00383g

**Published:** 2024-10-03

**Authors:** Finn L. Sebastian, Simon Settele, Han Li, Benjamin S. Flavel, Jana Zaumseil

**Affiliations:** a Institute for Physical Chemistry, Universität Heidelberg D-69120 Heidelberg Germany zaumseil@uni-heidelberg.de; b Department of Mechanical and Materials Engineering, University of Turku FI-20014 Turku Finland; c Turku Collegium for Science, Medicine and Technology, University of Turku FI-20520 Turku Finland; d Institute of Nanotechnology, Karlsruhe Institute of Technology D-76131 Karlsruhe Germany

## Abstract

Semiconducting single-wall carbon nanotubes (SWCNTs) are a promising material platform for near-infrared *in vivo* imaging, optical sensing, and single-photon emission at telecommunication wavelengths. The functionalization of SWCNTs with luminescent defects can lead to significantly enhanced photoluminescence (PL) properties due to efficient trapping of highly mobile excitons and red-shifted emission from these trap states. Among the most studied luminescent defect types are oxygen and aryl defects that have largely similar optical properties. So far, no direct comparison between SWCNTs functionalized with oxygen and aryl defects under identical conditions has been performed. Here, we employ a combination of spectroscopic techniques to quantify the number of defects, their distribution along the nanotubes and thus their exciton trapping efficiencies. The different slopes of Raman D/G^+^ ratios *versus* calculated defect densities from PL quantum yield measurements indicate substantial dissimilarities between oxygen and aryl defects. Supported by statistical analysis of single-nanotube PL spectra at cryogenic temperatures they reveal clustering of oxygen defects. The clustering of 2–3 oxygen defects, which act as a single exciton trap, occurs irrespective of the functionalization method and thus enables the use of simple equations to determine the density of oxygen defects and defect clusters in SWCNTs based on standard Raman spectroscopy. The presented analytical approach is a versatile and sensitive tool to study defect distribution and clustering in SWCNTs and can be applied to any new functionalization method.

## Abstract 2

Defect clustering in carbon nanotubes can be recognized by a new analytical approach combining quantum yield measurements and Raman spectroscopy.

## Graphical abstract

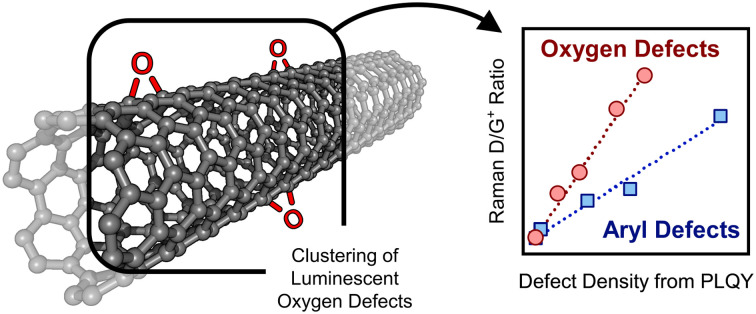

**New concepts**

A unique combination of spectroscopic techniques for the investigation of luminescent defects in single-wall carbon nanotubes (SWCNTs) reveals clustering of oxygen defects in contrast to aryl sp^3^ defects. The presented approach utilizes Raman spectroscopy and photoluminescence quantum yield (PLQY) measurements to elucidate the lateral distribution of different types of lattice defects along functionalized carbon nanotubes and the related efficiency of exciton trapping (independently corroborated by statistical analysis of single-SWCNT photoluminescence spectra at cryogenic temperatures). The quantitative comparison of Raman Δ(D/G^+^) ratios *versus* calculated defect densities from PLQY measurements enables the identification and characterization of defect clusters. This new method for the quantification of defect densities and defect distributions can be easily expanded to other functionalization methods. Thus, it is a powerful tool for the systematic investigation of luminescent defects in SWCNTs and for further optimization of their near-infrared emission.

## Introduction

The covalent functionalization of single-wall carbon nanotubes (SWCNTs) with luminescent defects has emerged as a promising strategy to improve and tune their near-infrared luminescence properties for optical sensing,^1–4^ high contrast *in vivo* imaging in the second biological window,^5,6^ high-purity single-photon emission,^7–9^ and optoelectronic devices operating at telecommunication wavelengths.^10–12^ Regardless of their exact chemical composition or binding configuration, luminescent defects act as zero-dimensional trap sites for mobile E_11_ excitons diffusing along the SWCNT lattice.^13,14^ Photoluminescence (PL) from these energetically lower states occurs at wavelengths even further red-shifted into the near-infrared (nIR) than E_11_ and with fluorescence lifetimes of hundreds of picoseconds. The energy difference between the mobile E_11_ exciton and defect emission (*i.e.*, optical trap depth, Δ*E*_opt_) ranges from 100 to 300 meV. Luminescent defects are able to trap and localize excitons at room temperature and thus prevent non-radiative decay at quenching sites (*e.g.*, nanotube ends). The suppression of this non-radiative decay path considerably enhances the photoluminescence quantum yields (PLQYs) of SWCNTs within a narrow window of optimal defect density.^15–18^ Most commonly these defects are created, characterized and theoretically investigated in chiral (6,5) carbon nanotubes due to their abundance, easy purification and high reactivity.^19–21^ Hence, we will focus on (6,5) SWCNTs from here on.

**Fig. 1:**
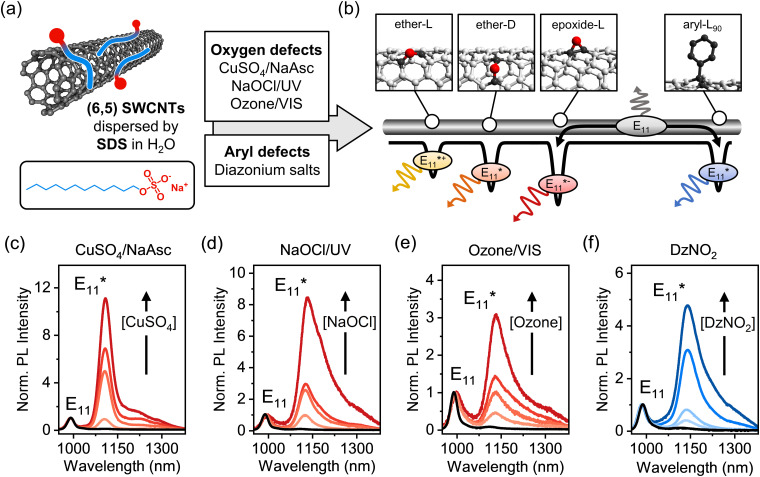
(a) Covalent functionalization of (6,5) SWCNTs with luminescent defects in aqueous dispersion with surfactant SDS. Oxygen defects are created using CuSO_4_/NaAsc, NaOCl/UV-light, or ozonation followed by SWCNT excitation with visible light. Treatment of SWCNTs with 4-nitrobenzenediazonium tetrafluoroborate (DzNO_2_) introduces aryl sp^3^ defects. (b) Schematic of different types of oxygen (ether-L, ether-D, and epoxide-L) and sp^3^ defects (aryl-L_90_) that act as trap sites with different depths for mobile E_11_ excitons. (c)–(f) Normalized (to E_11_) PL spectra of pristine (black) and functionalized (6,5) SWCNTs in aqueous dispersion.

Among the various types of luminescent defects in (6,5) SWCNTs, oxygen and aryl defects have been the main focus of experimental and theoretical studies, as they are easily introduced using reactive oxygen species (ROS)^6,20,22,23^ or aryldiazonium salts (Fig. 1a).^14,24–26^ In both cases, the emission wavelength of the defect PL is mainly determined by the binding configuration of the introduced defect moieties on the SWCNT lattice. For oxygen defects, three thermodynamically stable binding configurations can be distinguished.^13^ In one of them, the oxygen atom forms an epoxide, while the other two are ether-type configurations with different optical trap depths depending on the carbon–carbon bonds in either circumferential (ether-D) or longitudinal (ether-L) orientation with respect to the SWCNT axis, as shown schematically in Fig. 1b. Similar to that, two stable and experimentally observed configurations of sp^3^ defects with distinct PL emission wavelengths are created upon binding of aryl groups to the nanotube lattice and formation of two sp^3^ carbons in different *ortho* (*e.g.*, aryl-L_90_ see Fig. 1b) or *para* positions.^27–29^

Luminescent oxygen and aryl defects exhibit similar optical properties in addition to their equally red-shifted emission, such as nonlinear scaling of the defect emission intensity with excitation power^30^ and a significant energy offset between the optical and thermal trap depths.^31^ However, large deviations were found for the brightening factors for SWCNTs functionalized with oxygen or aryl sp^3^ defects. The reported absolute values vary, but, typically oxygen defects lead to an increase in total PLQY by a factor of 2–3,^6,20^ whereas aryl defects can cause a PLQY increase by a factor of >5, depending on the functional group and initial SWCNT length.^15,17,24^ A reversed order of bright and dark exciton states for oxygen and aryl defects was considered as a possible explanation.^14,31^ Additionally, the introduction of exciton quenching sites due to unintentional over-oxidation of SWCNTs under harsh conditions during functionalization with ROS may play a role.^6,20^ The efficient capture of mobile E_11_ excitons followed by defect emission depends on the distribution and trap depth of different defects on the nanotubes lattice, which is usually assumed to be uniform. However, clustering of defects may occur depending on the functionalization method and should have a substantial impact on emission properties relevant for applications in sensing, imaging and optoelectronics.

To this end, we present a comparative study of oxygen- and aryl-functionalized (6,5) SWCNTs and demonstrate how to recognize clustering of luminescent defects. We employ a combined analytical approach of Raman spectroscopy and spectrally-resolved PLQY measurements to quantify the absolute density of luminescent exciton quenching sites on the carbon nanotube lattice for different functionalization reactions. This method provides precise defect densities (per μm of nanotube) for aryl sp^3^ defects in different SWCNT species and is independent of the Raman excitation laser wavelength as shown previously.^32,33^ However, we find a striking difference in exciton trapping efficiency for SWCNTs with luminescent oxygen defects at identical levels of functionalization to aryl defects as corroborated by a statistical analysis of single-nanotube PL spectroscopy at cryogenic temperature. We attribute these findings to clustering of oxygen defects at length scales similar to or smaller than the mobile exciton size, whereas aryl defects act mostly as separate exciton traps. We propose the presented approach as a robust and powerful tool to identify and quantify inhomogeneities in defect distribution and defect clustering in functionalized SWCNTs.

## Results and discussion

To quantify and directly compare different luminescent oxygen and aryl defects, purified dispersions of (6,5) SWCNTs stabilized in water by sodium dodecyl sulfate (SDS) were obtained through aqueous two-phase extraction as reported previously.^32^ The nearly monochiral (6,5) SWCNT dispersions were characterized by UV-Vis-nIR absorption and PL excitation–emission spectroscopy (Fig. S1 and S2, ESI†). The functionalization of (6,5) SWCNTs with luminescent defects was performed using a variety of synthetic methods (see Fig. 1a). Oxygen defects were introduced *via* three previously reported procedures: ozonation of SWCNTs and subsequent photoconversion with visible light,^20^ UV-light driven dissociation of sodium hypochlorite (NaOCl),^6^ and with a Fenton-like reaction using copper(ii) sulfate (CuSO_4_) and sodium ascorbate (NaAsc)^23^ (for details, see Experimental methods, ESI†). While oxygen functionalization was shown to initially introduce ether-L, ether-D and epoxide-L defects with different trap depths (Fig. 1b),^13^ the epoxide defects mostly rearrange to the thermodynamically favored ether-D configuration upon exposure of SWCNTs to light.^23^ The covalent functionalization with aryl defects was accomplished using 4-nitrobenzenediazonium tetrafluoroborate (DzNO_2_), which forms radical species that attack the sp^2^-hybridized nanotube lattice leading to sp^3^ carbons with a predominantly aryl-L_90_ configuration.^28^

The degree of functionalization was tuned by varying the concentration of the respective reactants. In the case of SWCNT ozonation, the characteristic UV absorption peak of ozone at 260 nm was monitored to adjust its concentration in water prior to addition to the nanotube dispersion, as shown in Fig. S3, ESI.† Importantly, all functionalization reactions were performed on the same batch of SWCNTs and with the same surfactant (SDS) to ensure comparability of the subsequent characterization. The successful introduction of luminescent defects was confirmed by the emergence of defect-induced emission peaks at ∼1120 nm (ether-D defect) and ∼1140 nm (aryl-L_90_ defect) as shown in Fig. 1c–f (for absolute PL spectra, see Fig. S4, ESI†). These defect emission peaks are commonly labelled based on their spectral position with respect to the E_11_ emission and not their precise molecular origin. Hence, for both oxygen and aryl defects we refer to the main defect emission peaks as E_11_*. For all functionalization methods, a linear increase of the E_11_*/E_11_ PL intensity ratio with the reactant concentration was observed (Fig. S5, ESI†). Note that the Fenton-like reaction (Fig. 1c) leads to a narrower E_11_* emission peak, which we attribute to the very mild reaction conditions with fewer possible side-reactions.

**Fig. 2:**
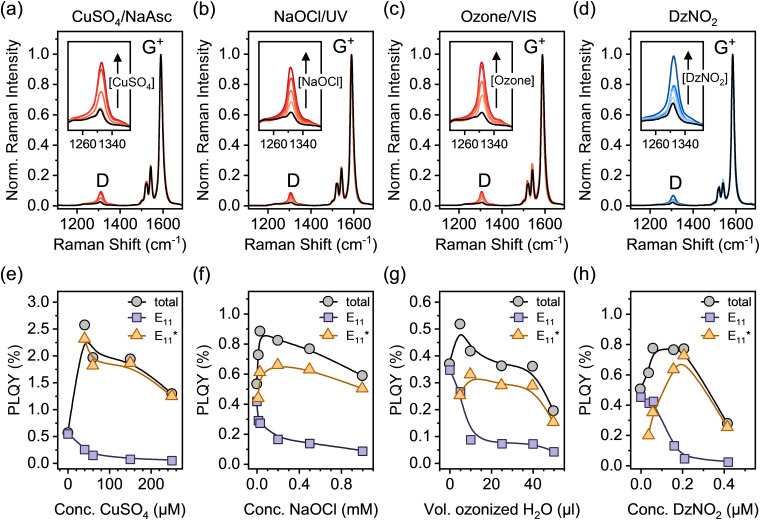
(a)–(d) Normalized (to G^+^ peak) averaged Raman spectra of pristine (black) and different functionalized (6,5) SWCNTs (*λ*_exc_ = 532 nm, >3600 individual spectra for each sample). The insets show the evolution of the defect-related D-mode with increasing degree of functionalization. (e)–(h) Corresponding total and spectrally resolved PLQYs for pristine and different functionalized (6,5) SWCNTs in aqueous dispersion depending on reactant concentration (after surfactant exchange to 1% (w/v) DOC).

After functionalization, a surfactant exchange of the SWCNTs to 1% (w/v) sodium deoxycholate (DOC) was carried out *via* spin-filtration to remove reaction by-products and generate stable dispersions for further analysis. Drop-cast films of all dispersions were analyzed by resonant Raman spectroscopy (laser excitation wavelength *λ*_exc_ = 532 nm). Fig. 2a–d show averaged Raman spectra for all functionalization methods at different defect densities (corresponding to samples in Fig. 1c–f). The Raman D-mode intensity is proportional to the number of point defects in the SWCNT lattice, whereas the intensity of the G^+^-mode originates from the longitudinal optical phonon of the sp^2^-hybridized nanotube lattice.^34^ Thus, the Raman D/G^+^ ratio increases with functionalization and can be used as a relative metric to estimate the density of luminescent defects. For different sp^3^ aryl defects in (6,5) SWCNTs – but not yet for oxygen defects – a quantitative correlation was established, *vide infra*.^32,33^

Treatment of nanotubes with oxidizing agents such as ozone, hypochlorite or CuSO_4_/NaAsc, although at low concentrations, can lead to slight p-doping, which is also reflected in their Raman spectra and may complicate the analysis for defect quantification. Signatures of doping, *i.e.*, increased intensities of the E_2_ mode as well as broadening of the G^+^- and 2D-mode linewidth were observed for some oxygen-functionalized SWCNTs.^35,36^ To separate these effects, films of pristine and functionalized (6,5) SWCNTs were intentionally p-doped by immersion in toluene solutions of the molecular oxidant F_4_TCNQ (for details, see Experimental methods, ESI†).^37,38^ While the typical signatures of p-doping are observed (Fig. S6, ESI†), no changes of the integrated Raman D/G^+^ ratio occur (Fig. S7, ESI†), indicating that low-level p-doping does not interfere with the defect quantification for oxygen-functionalized SWCNTs by Raman spectroscopy.

Complementary to the relative defect densities obtained by Raman spectroscopy, absolute defect densities can be calculated within the framework of the diffusion-limited contact quenching (DLCQ) model. Within this model, exciton diffusion in SWCNTs is assumed to be governed exclusively by radiative decay of highly mobile E_11_ excitons or non-radiative decay at quenching sites, *e.g.* nanotube ends.^17,39^ Covalent functionalization with luminescent defects introduces new loss channels for E_11_ excitons, leading to an additional decrease of the E_11_ PLQY. This enables the calculation of luminescent defect densities, provided that PLQYs can be determined reliably. The combination of relative Raman D/G^+^ ratios and defect densities calculated from the spectrally resolved E_11_ PLQY of pristine and functionalized SWCNTs is a powerful tool to compare and investigate different types of luminescent defects as both techniques are highly sensitive to small changes in the electronic structure of nanotubes. For sp^3^ aryl defects created by different synthetic routes this method was already shown to provide a robust correlation for defects densities up to ∼40 μm^−1^ and thus enables facile quantification of the defect density by Raman spectroscopy alone.^32,33^

To compare and expand this correlation to oxygen defects, we determined the absolute PLQYs of (6,5) SWCNTs functionalized with oxygen and aryl defects and calculated the defect densities from the measured decrease in E_11_ PLQY (for details on PLQY measurements and defect density calculation see Experimental methods, ESI†). Spectrally resolved PLQYs for pristine and functionalized SWCNTs are presented in Fig. 2e–h. At low degrees of functionalization, an increase of the total PLQY is observed irrespective of the functionalization method. Efficient exciton trapping and radiative relaxation *via* E_11_* emission overcompensates for the simultaneous decrease in E_11_ emission. At medium to high levels of functionalization, the total PLQY decreases again, as the defects start to significantly disturb the electronic structure of the pristine SWCNT lattice.

**Fig. 3:**
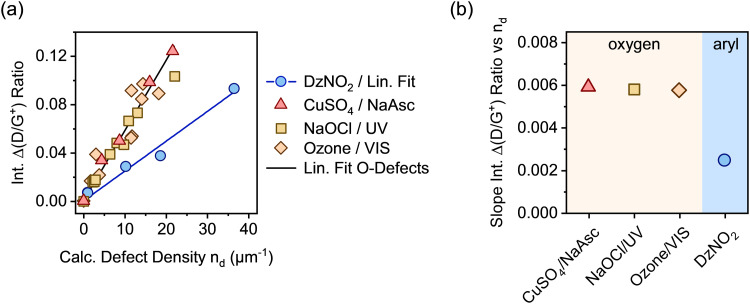
(a) Integrated Raman Δ(D/G^+^) ratios *versus* calculated defect densities (*n*_d_) based on PLQY for (6,5) SWCNTs functionalized by different methods creating oxygen or aryl sp^3^ defects. Linear fits for oxygen defects, combined (*R*^2^ = 0.97) and aryl sp^3^ defects (*R*^2^ = 0.98). (b) Extracted slopes of the integrated Δ(D/G^+^) ratio *versus* calculated defect density for each functionalization method.

In analogy to our previous studies on aryl defects,^32,33^ the change of the integrated Raman D/G^+^ ratios (Δ(D/G^+^)) was correlated with the calculated defect densities *n*_d_ from E_11_ PLQY measurements (see Fig. 3a). Using the Δ(D/G^+^) ratio instead of the absolute values generally ensures higher comparability of the Raman data across different SWCNT batches and starting materials. In this work, the same batch of nanotube material was used for all samples. A linear dependence of the Δ(D/G^+^) ratio on the calculated density of luminescent oxygen defects was obtained for all samples. For luminescent aryl defects, the slope was the same as previously reported for (6,5) SWCNTs.^32,33^ In contrast to that, a strong deviation from this gradient is evident for all oxygen-functionalized nanotubes. Regardless of the functionalization method, all samples of (6,5) SWCNTs with luminescent oxygen defects exhibit a slope that is larger by a factor of ∼2.3 than that for the aryl defects (see Fig. 3b; for individual linear fits see Fig. S8, ESI†). Similar differences are also observed for other defect-related Raman peaks, such as the intermediate frequency modes (IFMs)^33,40^ presented in Fig. S9–S11, ESI.†

Clearly, despite very similar spectral signatures in PL and Raman spectroscopy, there must be a fundamental difference between luminescent oxygen and aryl defects in SWCNTs and their interaction with mobile excitons. Importantly, the calculation of defect densities based on the DLCQ model only considers the diffusion constant and radiative lifetime of the E_11_ exciton but no defect-specific photophysical processes.^17,41^ Hence, it gives direct access to the E_11_ exciton quenching probability of local defects on the SWCNT lattice. However, the observed deviation of the slopes of the Raman D/G^+^ ratios indicate either a different D-mode Raman cross-section or higher defect densities for oxygen-functionalized compared to aryl-functionalized nanotubes.

Previous work on defective carbon-based materials indicated that the intensity of the disorder-related Raman D-mode is not particularly sensitive to the actual structure (functional group or hybridization) of point-like lattice defects.^34,42^ As all types of oxygen and aryl defects involve binding to exactly two carbon atoms, the defect size and thus the probability of second-order scattering processes (*i.e.*, Raman D-mode cross-section) should be equally insensitive to the precise chemical nature of the point-like defects. This assumption only leaves a higher number of structural defects than calculated from PLQY measurements as a possible explanation, which could be caused by clustering of the oxygen defects. The possibility of clustering of defects during covalent functionalization of carbon nanotubes was considered before,^43^ but only shown to occur in additional alkylation reactions on already functionalized SWCNTs.^44^ Furthermore, theoretical studies on SWCNT shortening by chemical oxidation with hydrogen peroxide suggest that ROS preferentially attack the carbon nanotube lattice at already defective areas, which may promote clustering.^45^

For a cluster of defects with an average spacing similar to or smaller than the size of the exciton (*i.e.*, few nanometers^17,46^), several defects may act as a single quenching site for the mobile excitons in the DLCQ model. Furthermore, collective exciton states of coupled defects were proposed for closely spaced luminescent defects.^47–50^ Irrespective of the actual local electronic structure, the exciton trapping efficiency probably does not simply scale linearly with the absolute number of individual luminescent defects. Their precise distribution along the SWCNT lattice will strongly influence the correlation between lattice defects as determined from the Raman D/G^+^ ratio and the calculated defect density from E_11_ PLQY measurements.

To directly assess the possibility of defect clustering for oxygen defects, we performed PL spectroscopy on individual oxygen-functionalized SWCNTs embedded in a polymer matrix at cryogenic temperatures (4.7 K). Under these conditions SWCNTs show strong localization of even the E_11_ excitons due to slight variations in the local dielectric environment.^51,52^ Luminescent defects are similarly influenced by dielectric fluctuations of the environment. Thus, the defect emission energies even of luminescent defects with identical binding configuration vary slightly. These differences can be resolved at cryogenic temperatures due to the strongly reduced linewidths. Hence, it is possible to quantify the number of luminescent defects on a single nanotube by simply counting the different peaks, as demonstrated previously for aryl defects in polymer-wrapped SWCNTs.^32^ The statistical analysis of the number of defect emission peaks provides direct access to the average number of individual emissive sites per nanotube. Together with atomic force microscopy (AFM) to determine the average nanotube length, the density of luminescent defects per μm can be obtained and compared to the results obtained from PLQY measurements and the DLCQ model.

**Fig. 4:**
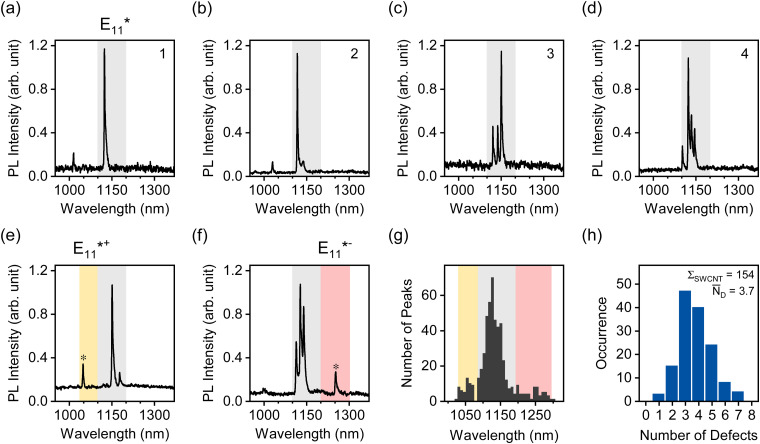
(a)–(d) Low-temperature (4.7 K) PL spectra of individual (6,5) SWCNTs functionalized with CuSO_4_/NaAsc, embedded in a polystyrene matrix. The number of oxygen defect sites per nanotube (top right corner) was determined as the number of peaks in the spectral region of the E_11_* emission (grey area). (e) and (f) Representative low-temperature spectra where PL emission from the E_11_*^+^ (yellow area) and E_11_*^−^ (pink area) defect configurations is observed. (g) Spectral distribution of all counted defect PL peaks. (h) Defect PL peak histogram of 154 individual (6,5) SWCNTs with an average defect density of 3.7 defects per nanotube.

To perform this analysis, oxygen-functionalized (6,5) SWCNTs were transferred to toluene solutions of the fluorene–bipyridine copolymer PFO-BPy, which exhibits a high dispersion efficiency for (6,5) SWCNTs.^21^ After transfer of highly dilute nanotube dispersions into a polystyrene matrix, single-nanotube PL spectra were acquired at 4.7 K in an optical cryostat (for details see Experimental methods, ESI†). Representative PL spectra for individual (6,5) SWCNTs with E_11_* oxygen defects are displayed in Fig. 4a–d. Single defects can be identified as narrow peaks in the respective spectral region characteristic for a certain binding configuration (1030–1100 nm for E_11_*^+^, 1100–1200 nm for E_11_*, 1200–1300 nm for E_11_*^−^). Fig. S12 and S13 (ESI†) show additional single-SWCNT PL spectra.

About 30% of the measured SWCNTs exhibited defect PL emission in the E_11_*^+^ and E_11_*^−^ spectral region (Fig. 4e–g). The E_11_*^+^ emission likely occurs from longitudinally oriented ether-type defects (ether-L) with a shallower exciton trap depth (Δ*E*_opt_ ∼ 50 meV, compared to ∼140 meV for E_11_* and ∼250 meV for E_11_*^−^).^13^ This defect is also often referred to as the Y_1_ band in PL spectra of SWCNTs and becomes more prominent in thin films of SWCNTs on substrates with polar surfaces (*e.g.*, SiO_2_ or glass) upon annealing.^53^ Emission from this shallow exciton trap state is low at room temperature but becomes significant under cryogenic conditions. It is plausible that thermal detrapping of excitons from these shallow traps back to the E_11_ level is dominant at room temperature.^31^ Hence, E_11_*^+^ defects should not contribute to the decrease in E_11_ PLQY to the same degree as deeper exciton traps (E_11_* and E_11_*^−^) while still leading to a higher Δ(D/G^+^) ratio.

**Fig. 5:**
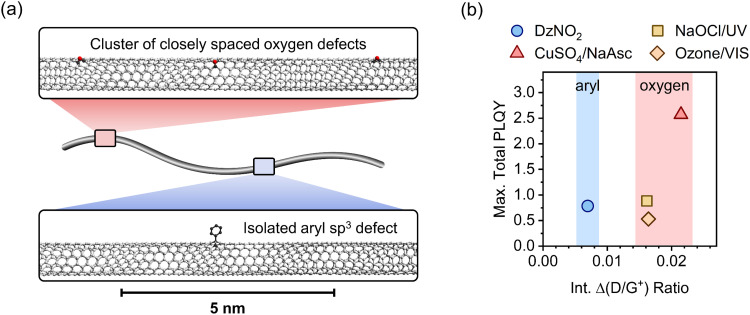
(a) Schematic depiction of defect clustering in SWCNTs. Closely spaced (smaller than the size of a mobile exciton) luminescent oxygen defects act as one effective exciton trap compared to individual aryl sp^3^ defects while contributing more to the Raman D/G^+^ ratio. (b) Maximum total PLQYs *versus* integrated Δ(D/G^+^) ratio for different functionalization methods. For all luminescent oxygen defects, the highest PLQYs are obtained for larger D/G^+^ ratios compared to aryl sp^3^ defects.

A full statistical analysis of over 150 single-SWCNT PL spectra gave an average value of 3.7 luminescent defects per nanotube, with a minimum of one and a maximum of seven defects per nanotube, as shown in Fig. 4h. AFM length statistics of the sample yielded an average nanotube length of 310 nm (compare Fig. S14, ESI†). Combining these values gives an average defect density of 11.9 μm^−1^ for oxygen-functionalized SWCNTs, which is 2.4 times larger than the calculated defect density of 4.9 μm^−1^ from the E_11_ PLQY of the same sample. When the same counting method was applied to sp^3^ aryl defects in previous studies, the defect densities aligned remarkably well with the numbers extracted with the DLCQ model.^32^ This ratio of real and expected defect density is also remarkably close to the factor of 2.3 by which the Δ(D/G^+^) *vs. n*_d_ slope deviates for oxygen and aryl defects as shown in Fig. 3a.

Clearly, the correlation between the Raman Δ(D/G^+^) ratio and the defect density – determined from E_11_ PLQYs or by counting the defects on individual SWCNTs – is strikingly different for oxygen and aryl sp^3^ defects. As argued above, this deviation could be explained with the preferential formation of oxygen defect clusters by ROS-based functionalization methods. Assuming that 2 to 3 defects form a cluster smaller than the size of a mobile exciton, these excitons would interact with such clustered oxygen defects as one collective trap state at room temperature. This would result in an apparently lower trapping efficiency per structural defect as measured by the Raman D/G^+^ ratio. Thus, higher E_11_ PLQYs and lower calculated defect densities would be observed for the same number of structural defects. Conversely, at cryogenic conditions even minor differences in the local dielectric environment of oxygen defect clusters due to inhomogeneities of the polymer matrix would distinguish these traps from each other and enable a direct quantification. At low temperatures, exciton detrapping is suppressed, and even individual defects with low exciton trapping probabilities within a larger cluster can be identified in single-nanotube PL spectra.

The remarkable agreement between data obtained independently by Raman/PLQY and cryo-PL/AFM measurements suggests that the quantification of the number of oxygen defect clusters can be performed by adapting the equation derived for aryl defects.^32^ The number of collective exciton traps in the form of oxygen clusters is then1
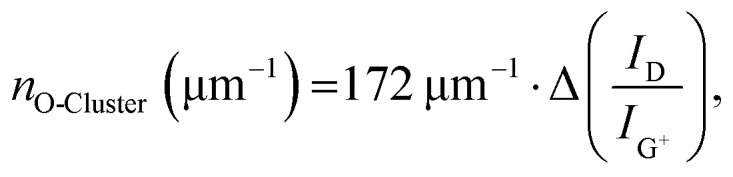
where Δ(*I*_D_/*I*_G^+^_) represents the change of the integrated Raman D/G^+^ ratio of the functionalized (6,5) SWCNT sample compared to the pristine samples (for resonant excitation at 532 nm). Consequently, the actual number of individual oxygen atom defects can be calculated with2
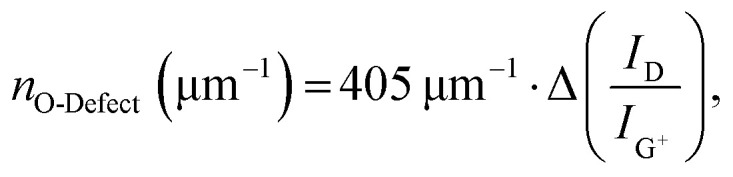
which is – within the margin of experimental error – identical to the previously reported equation for individual aryl sp^3^ defects.^32^

Fig. 5a gives a schematic representation of luminescent oxygen and aryl defects in SWCNTs. Corresponding to the deviation in Raman Δ(D/G^+^) ratio *vs*. *n*_d_ slopes, one oxygen defect cluster should correspond to 2–3 individual oxygen defects. This structural model provides a straightforward tool to assess already developed, and in particular, new functionalization methods. If the correlation of Raman Δ(D/G^+^) ratios and calculated defect densities results in values smaller than 172 μm^−1^ (compare eqn (1)), stronger clustering should be expected compared to oxygen defects. Likewise, larger values are an indicator for wider separation of individual structural defects.

For the application of functionalized SWCNTs as emitters the question arises whether defect clustering is desirable or detrimental for reaching the highest total PLQY. Hence, we correlated the maximum total PLQYs for the different functionalization methods with the corresponding Raman Δ(D/G^+^) ratios (see Fig. 5b). Interestingly, the maximum PLQY for SWCNTs with aryl sp^3^ defects occurs at significantly lower degrees of functionalization compared to oxygen defects. A higher degree of functionalization is required to obtain the brightest SWCNTs with oxygen defects. This might be attributed to the higher actual number of single oxygen defects necessary to achieve the optimal exciton trapping efficiency. The number of oxygen clusters at which maximum total PLQYs are observed are in good agreement with the density of luminescent exciton traps necessary to achieve optimal brightening for aryl-functionalized SWCNTs (∼4–8 defects μm^−1^). Interestingly, the maximum PLQY (2.5%) for SWCNTs functionalized with the Fenton-like reaction is substantially higher than those for all other methods (0.5–0.9%). This surprising difference, especially compared to the other oxygen functionalization methods, might be rationalized with the continuous production of low concentrations of ROS by the Fenton-like reaction (with CuSO_4_/NaAsc) and thus a more controlled reaction with the nanotube lattice. In contrast to that, the harsh conditions of the other methods (*e.g.*, treatment with ozone, hypochlorite or other inorganic oxidizing agents) are more likely to create higher concentrations of ROS and thus may lead to over-functionalized areas that act only as quenching sites and may also cause the observed broadening of the defect emission peaks.^6,54^

## Conclusion

In summary, we have shown that the combination of Raman spectroscopy, spectrally-resolved PLQY measurements, and low-temperature single-nanotube PL spectroscopy can be used to recognize and quantify clustering of luminescent oxygen defects in functionalized (6,5) SWCNTs in comparison to typical aryl sp^3^ defects. One oxygen defect cluster contains about 2–3 individual defects that act as one exciton trap. Since clustering of oxygen defects occurs irrespective of the method of functionalization, we can provide simple and general equations for the precise quantification of oxygen defects and oxygen defect clusters in (6,5) SWCNTs based on standard resonant Raman spectroscopy alone. We show that the comparison of the slopes of the Raman Δ(D/G^+^) ratios vs calculated defect densities from PLQY measurements can serve as a direct measure for clustering. This quantification method can be expanded easily to other functionalization protocols and gives indirect information about the local distribution of exciton trapping sites and individual defects. Hence, this comparative study provides a blueprint for the characterization of new synthetic methods for the introduction of luminescent defects. For example, the functionalization of SWCNTs with bidentate reactants such as bisdiazonium compounds^25^ or divalent functional groups^55^ could be investigated in more detail based on this approach. A comparison with the reference slopes presented here should enable a quick and reliable assessment of possible defect clustering. Overall, the absolute quantification of defect densities by combining Raman spectroscopy and PLQY measurements is a valuable tool for the systematic and reproducible investigation of luminescent defects in SWCNTs.

## Author contributions

F. L. S. fabricated and measured all samples and analyzed the data. S. S. contributed to sample preparation and characterization. H. L. and B. S. F. provided ATPE-sorted (6,5) SWCNTs. J. Z. conceived and supervised the project. F. L. S. and J. Z. wrote the manuscript with input from all authors.

## Data availability

The data generated and analyzed for this article are available in the heiDATA repository at https://doi.org/10.11588/data/TS6XA6.

## Conflicts of interest

The authors declare no competing interests.

## Supplementary Material

NH-009-D4NH00383G-s001
