# Supplementary material for: How to recognize clustering of luminescent defects in single-wall carbon nanotubes
Source: Nanoscale Horiz. 2024 Oct 3;9(12):2286–94. doi: 10.1039/d4nh00383g (PMC11462117; doi:10.1039/d4nh00383g)
Supplement: NH-009-D4NH00383G-s001 [file NH-009-D4NH00383G-s001.pdf]

Supplementary Information (SI) for Nanoscale Horizons.
This journal is © The Royal Society of Chemistry 2024

Electronic Supplementary Information (ESI)

How to Recognize Clustering of Luminescent Defects in Single-Wall Carbon Nanotubes

Finn L. Sebastian[1], Simon Settele[1], Han Li[2,3], Benjamin S. Flavel[4], and Jana Zaumseil[1,*]

[1]Institute for Physical Chemistry, Universität Heidelberg, D-69120 Heidelberg, Germany

[2]Department of Mechanical and Materials Engineering, University of Turku, FI-20014 Turku, Finland

[3]Turku Collegium for Science, Medicine and Technology, University of Turku, FI-20520 Turku, Finland

[4]Institute of Nanotechnology, Karlsruhe Institute of Technology, D-76131 Karlsruhe, Germany

Corresponding Author

*E-mail: zaumseil@uni-heidelberg.de

Experimental Methods......S-3
Dispersion and selection of (6,5) SWCNTs S-3
Functionalization of (6,5) SWCNTs with oxygen and 4-nitroaryl defects......S-3
Chemical doping of (6,5) SWCNTs films S-4
Transfer of aqueous (6,5) SWCNT dispersions to toluene......S-4
Characterization......S-5
Photoluminescence quantum yields......S-5
Calculation of luminescent defect density......S-6
Single-SWCNT PL spectroscopy at cryogenic temperatures......S-7
Additional spectra S-8
UV-Vis-nIR absorption spectrum of pristine (6,5) SWCNTs S-8
Photoluminescence excitation-emission map of pristine (6,5) SWCNTs S-8
UV-Vis absorption spectrum of ozonated water S-9
Absolute PL spectra for different functionalization methods......S-10
E_{11}*/E_{11} PL intensity ratios for different functionalization methods S-11
Raman spectra of p-doped pristine and functionalized SWCNTs......S-12
Raman Δ(D/G$^+$) *vs* calculated defect density n_d for different functionalization methods .S-14
Raman spectroscopy of pristine and functionalized SWCNTs (RBM and IFM region)...S-15
Raman Δ(IFM/RBM) *vs* calculated defect density n_d for different functionalization methods S-17
Extracted Raman Δ(IFM/RBM) *vs* n_d slopes......S-18
Additional low-temperature single-SWCNT PL spectra......S-19
AFM length statistics of functionalized SWCNTs......S-21
References S-22

Experimental Methods

Dispersion and selection of (6,5) SWCNTs

(6,5) SWCNTs were selected from CoMoCAT raw material (CHASM SG65i-L58) by aqueous two-phase extraction (ATPE) as reported previously.[1] The raw material was dispersed in deoxycholate (DOC, BioXtra) and transferred to a two-phase system of dextran (M_W = 70 kDa, TCI Chemicals) and poly(ethylene glycol) (PEG, M_W = 6 kDa, Alfa Aesar). For separation of (6,5) SWCNTs, a diameter sorting protocol based on addition of sodium dodecyl sulfate (SDS, Sigma-Aldrich) was employed. The concentration of SDS was increased to 1.1% (w/v) at a fixed DOC concentration of 0.04% (w/v) for removal of large diameter SWCNTs from the top phase. (6,5) SWCNT-enriched fractions were collected between SDS concentration of 1.2% to 1.5% (w/v). Semiconducting and metallic SWCNTs were separated by addition of sodium cholate (SC, Sigma-Aldrich) and sodium hypochlorite (NaOCl, Sigma-Aldrich) as an oxidant. The selected (6,5) SWCNTs in DOC were concentrated by filtration in a pressurized ultrafiltration stirred cell (Millipore) with a M_W = 300 kDa cut-off membrane, and 1% (w/v) SDS was added before further use.

Functionalization of (6,5) SWCNTs with oxygen and 4-nitroaryl defects

For the functionalization of (6,5) SWCNTs with oxygen defects, three different methods were used. The first method employed a Fenton-like reaction based on the addition of copper(II) sulfate pentahydrate ($CuSO_4(H_2O)_5$, Sigma-Aldrich, ≥99.9% trace-metal basis) and sodium-L-ascorbate (NaAsc, Sigma-Aldrich, ≥99%) according to a procedure by Settele *et al.*[2] In short, aqueous dispersions of (6,5) SWCNT were adjusted to an optical density (OD) of 0.33 cm^{-1} at the E_{11} absorption peak with ultra-pure water (total volume 1 ml), and a final surfactant concentration of 0.33% (w/v) SDS. Aliquots of aqueous solutions of $CuSO_4$ and NaAsc were added to (6,5) SWCNT dispersions to achieve final concentrations of $CuSO_4$ between 25 and 250 µM and a molar ratio of 1:12 ($CuSO_4$:NaAsc) in the reaction mixture. After storage in the dark for 16 h, the reaction was stopped by addition of Na_4EDTA (10 µl, 1.4 M, Sigma-Aldrich, 98%) and 10% (w/v) DOC (20 µl), followed by irradiation with UV-light for 2 h (365 nm, SOLIS-365C, Thorlabs, 1.9 mW·mm^{-2}) to promote the reorganization of oxygen defects.

The second functionalization method was adapted from a protocol reported by Lin *et al.*[3] ATPE-sorted dispersions of (6,5) SWCNTs in 1% (w/v) SDS were diluted to an OD of 0.1 cm^{-1} (total volume 3 ml) and a final SDS concentration of 0.1% (w/v). NaOCl (12% available chloride, Carl Roth) was added to achieve final concentrations between 0.015 and 1 mM in the reaction

mixture. After irradiation with UV-C-light (254 nm, VL-215.LC, Vilber Lourmat, 15 W) for 30 min, the reaction was stopped by addition of 20 µl of 10% (w/v) aqueous DOC solution.

The third functionalization method was adapted from Ghosh *et al.*[4] Ozone (Ozone Generator, Cambridge NanoTech Inc.) was bubbled through 7 ml of ultra-pure water in a Schlenk tube for 10 min, followed by dilution to an OD of 0.3 cm^{-1} at the ozone UV absorption peak (260 nm). ATPE-sorted dispersions of (6,5) SWCNTs were adjusted to an OD of 0.2 cm^{-1} (total volume 1.5 ml) and a final SDS concentration of 0.2 cm^{-1}. Depending on the intended degree of functionalization, between 5 and 50 µl of the ozonated ultra-pure water were slowly added while stirring. Photoconversion of the reaction product was achieved by irradiation with a green light-emitting diode (525 nm, SOLIS-525C, Thorlabs, 1.5 mW·mm^{-2}) until no further changes of the PL spectrum were observed (~ 60-90 min).

For functionalization of SWCNTs with 4-nitroaryl defects, ATPE-sorted (6,5) SWCNTs were diluted to an OD of 0.33 cm^{-1} at the E_{11} absorption peak (total volume 1 ml) and an SDS concentration of 0.33 cm^{-1}. Aliquots of aqueous solutions of 4-nitrobenzenediazonium tetrafluoroborate ($DzNO_2$, TCI Chemicals, >98%) were added to achieve concentrations between 0.04 and 0.42 µM in the reaction mixture. After storage in the dark for 7 days to ensure complete decomposition of the diazonium salt, 20 µl of 10% (w/v) aqueous DOC solution were added to the reaction mixture.

Independent of the functionalization method, SWCNT dispersions were spin-filtrated (Amicon Ultra-4, 100 kDa cut-off) immediately after completion of the reaction and transferred to 1% (w/v) aqueous DOC before further characterization.

Chemical doping of (6,5) SWCNTs films

Drop-cast films of (6,5) SWCNTs on glass (Schott AF32eco) were immersed in solutions of the molecular p-type dopant 2,3,5,6-tetrafluoro-7,7,8,8-tetracyanoquinodimethane (F_4TCNQ, TCI Chemicals, >98%) in toluene (200 µg·ml^{-1}) for different periods of time. The films were rinsed with toluene and blow-dried with nitrogen before further characterization.

Transfer of aqueous (6,5) SWCNT dispersions to toluene

A volume of 5 ml of functionalized (6,5) SWCNTs in 0.33% (w/v) aqueous SDS solution were filtered through PTFE membranes (Merck Millipore JVWP, pore size 0.1 µm) and washed thoroughly with ultra-pure water to remove the surfactant. The filter was then subjected to bath sonication for 30 min in a solution of poly[(9,9-dioctylfluorenyl-2,7-diyl)-*alt*-(6,6′-(2,2′-bipyridine))] (PFO-BPy, American Dye Source, M_W = 40 kDa, 0.5 g·L^{-1}) in toluene (5 ml, Sigma-

Aldrich, ≥99.5%). Unexfoliated material was removed by centrifugation of the stock dispersion, followed by centrifugation of the supernatant (each step at 60000 g for 45 min, Beckman Coulter Avanti J26XP centrifuge). The supernatant obtained after the second step was used to prepare samples for AFM and low-temperature PL spectroscopy.

Characterization

Baseline-corrected UV-Vis-nIR absorption spectra were recorded with a Cary 6000i absorption spectrometer (Varian, Inc.). A scattering background $S(\lambda) = S_0 e^{-b\lambda}$ was subtracted from the acquired spectra.[5]

Atomic force microscopy (AFM) was performed under ambient conditions with a Bruker Dimension Icon atomic force microscope in ScanAsyst mode. The SWCNTs were spin-coated (2000 rpm, 60 s) onto cleaned native silicon wafers from dispersions obtained after transfer of SDS-dispersed SWCNTs to toluene/PFO-BPy, followed by rinsing with tetrahydrofuran (THF) and isopropanol (IPA). Gwyddion 2.64 was used for nanotube length distribution analysis.

Raman spectra of pristine and functionalized (6,5) SWCNTs were acquired with a Renishaw InVia Reflex confocal Raman microscope in backscattering configuration (50× long working distance objective, Olympus, N.A. 0.5) under near-resonant excitation with a 532 nm laser. Dispersions of SWCNTs were drop-cast onto glass substrates (Schott AF32eco), and gently rinsed with water. Over 3600 spectra were collected and averaged for each sample. Integrated Raman $\Delta(D/G^+)$ ratios were determined by integration of the average normalized Raman spectra from 1200 – 1400 cm^{-1} (D-mode) and 1560 – 1650 cm^{-1} (G^+-mode).

Room-temperature photoluminescence (PL) spectroscopy and emission-excitation (PLE) mapping was performed with a Fluorolog-3 spectrometer (Horiba Jobin-Yvon). A xenon arc-discharge lamp (450 W) coupled to a double grating monochromator was employed as the excitation light source. Light emitted from the SWCNT dispersions was passed through a long-pass filter (850 nm), Czerny-Turner imaging spectrograph and recorded with a liquid nitrogen cooled InGaAs line camera (Symphony II).

Photoluminescence quantum yields

Absolute photoluminescence quantum yields (PLQYs) of pristine and functionalized dispersions were determined with an integrating sphere as the ratio of emitted (N_{em}) and absorbed photons (N_{abs}) in accordance with previously reported procedures:[6, 7]

$$\eta = \frac{N_{em}}{N_{abs}} \tag{1}$$

SWCNT dispersions were diluted to an OD of 0.15 to 0.2 cm^{-1} at the E_{11} transition to minimize re-absorption. A quartz glass cuvette (Hellma Analytics QX) with 1 ml of dispersion was placed in an integrating sphere (LabSphere, Spectralon coating). The spectrally filtered output of a picosecond-pulsed supercontinuum laser (NKT Photonics SuperK Extreme) was used for resonant excitation of SWCNTs at the E_{22} transition (570 nm), and the light exiting the integrating sphere was coupled into the spectrometer (Acton SpectraPro SP2358) with an optical fiber. The PL of the sample in the near-infrared (nIR) and the attenuated laser signal at the excitation wavelength were recorded with a liquid nitrogen-cooled InGaAs line camera (Princeton Instruments OMA V:1024). Identical measurements were performed with 1% (w/v) aqueous DOC to correct for light absorption and scattering of the solvent. Integration of the sample emission yields a value proportional to the number of emitted photons. Similarly, the integrated attenuated laser signal for each sample is subtracted from the value obtained for the solvent reference to determine the number of absorbed photons. To account for the wavelength-dependent sensitivity of the detector and the absorption of optical components, a stabilized broadband light source (Thorlabs SLS201/M, 300-2600 nm) with known spectral output was used to collect calibration lamp spectra.

Calculation of luminescent defect density

Luminescent defect densities were calculated based on the model of diffusion-limited contact quenching (DLCQ) of excitons in SWCNTs as reported previously.[1] The DLCQ model assumes that E_{11} excitons undergo either nonradiative quenching at quenching sites, *e.g.*, nanotube ends, or decay *via* radiative E_{11} PL emission. The PLQY of the E_{11} emission in pristine SWCNT can be calculated as

$$\eta = \frac{\pi}{2 \cdot n_q^2 \cdot D \cdot \tau_{rad}}\ , \tag{2}$$

where n_q represents the number of quenching sites per µm of nanotube, D is the exciton diffusion constant and τ_{rad} the radiative lifetime of the E_{11} exciton. Here, values of $D = 10.7 \pm 0.4\ cm^2{\cdot}s^{-1}$ and $\tau_{rad} = 3.35 \pm 0.41$ ns are used as reported in previous experimental studies.[8, 9] The introduction of luminescent defects (n_d) leads to additional radiative decay pathways resulting in a reduction of the E_{11} PLQY η^* of functionalized SWCNTs:

$$\eta^* = \frac{\pi}{2 \cdot (n_q + n_d)^2 \cdot D \cdot \tau_{rad}} \tag{3}$$

The combination of equations (2) and (3) yields an expression for the number density (µm^{-1}) of luminescent defects:

$$n_d = n_q\left(\sqrt{\frac{\eta}{\eta^*}} - 1\right) = \sqrt{\frac{\pi}{2 \cdot \eta \cdot D \cdot \tau_{rad}}}\left(\sqrt{\frac{\eta}{\eta^*}} - 1\right) \tag{4}$$

Single-SWCNT PL spectroscopy at cryogenic temperatures

Dispersions obtained after transfer of SDS-dispersed SWCNTs to toluene/PFO-BPy were diluted to an OD of 0.005 cm^{-1} at the E_{11} absorption peak and mixed with a solution of polystyrene (Polymer Source Inc., M_W = 230 kDa) in toluene (40 g·L^{-1}) in equal volumes. Spin-coating (2000 rpm, 60 s) of the obtained mixture (30 µl) onto gold-coated (150 nm) glass substrates completed the sample preparation.

A closed-cycle liquid helium cooled optical cryostat (Montana Instruments, Cryostation s50) was employed to perform low-temperature single-SWCNT PL spectroscopy at 4.7 K. To this end, a nIR-optimized ×50 long-working distance objective (Mitutoyo, N.A. 0.42) was used to focus the output of a continuous wave laser diode (Coherent, Inc. OBIS 640 nm, 1 mW) onto the sample. Emitted light was collected and projected through a long-pass filter (850 nm) onto a grating spectrograph (Princeton Instruments, IsoPlane SCT-320) equipped with a 1200 nm blaze grating (85 lines per mm). PL spectra were recorded with a thermoelectrically cooled two-dimensional InGaAs camera array (Princeton Instruments, NIRvana 640ST). The data collection was automated using a custom script to control a piezo-based nanopositioning system (Attocube ANC350, ANPx101 and ANPz102 nanopositioners). A small number of PL spectra probably originating from bundled or aggregated SWCNTs were identified by the presence of broad and ill-defined E_{11} PL emission in accordance with literature reports.[1,10,11] These spectra were disregarded in the subsequent statistical analysis.

Additional spectra

UV-Vis-nIR absorption spectrum of pristine (6,5) SWCNTs

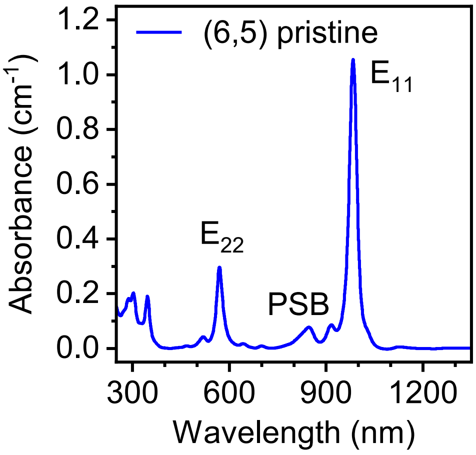

Figure S1. Baseline-corrected UV-Vis-nIR absorption spectrum of pristine (6,5) SWCNTs in aqueous dispersion (1% (w/v) SDS), with assignment of the E_{22}, E_{11}, and E_{11} phonon sideband (PSB) optical transitions.

Photoluminescence excitation-emission map of pristine (6,5) SWCNTs

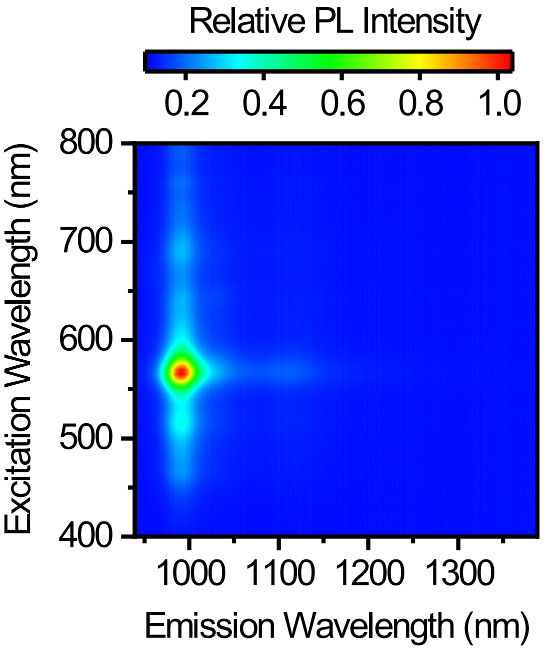

Figure S2. Photoluminescence excitation-emission map of pristine (6,5) SWCNTs in aqueous dispersion (0.3% (w/v) SDS).

UV-Vis absorption spectrum of ozonated water

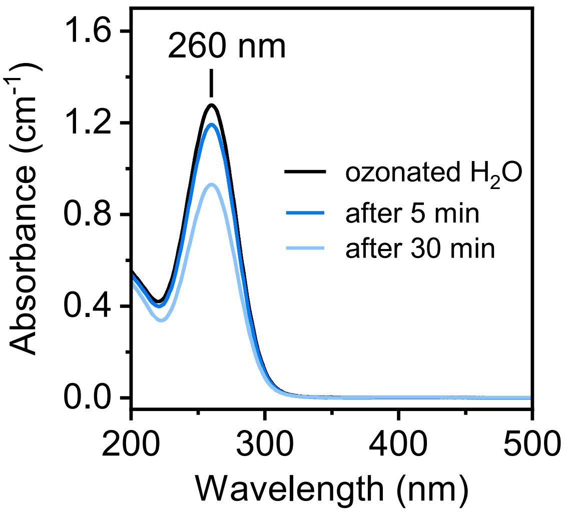

Figure S3. UV-Vis-nIR absorption spectra of water after ozonation, demonstrating the evolution of the ozone absorption peak (260 nm) 5 min and 30 min after ozonation.

Absolute PL spectra for different functionalization methods

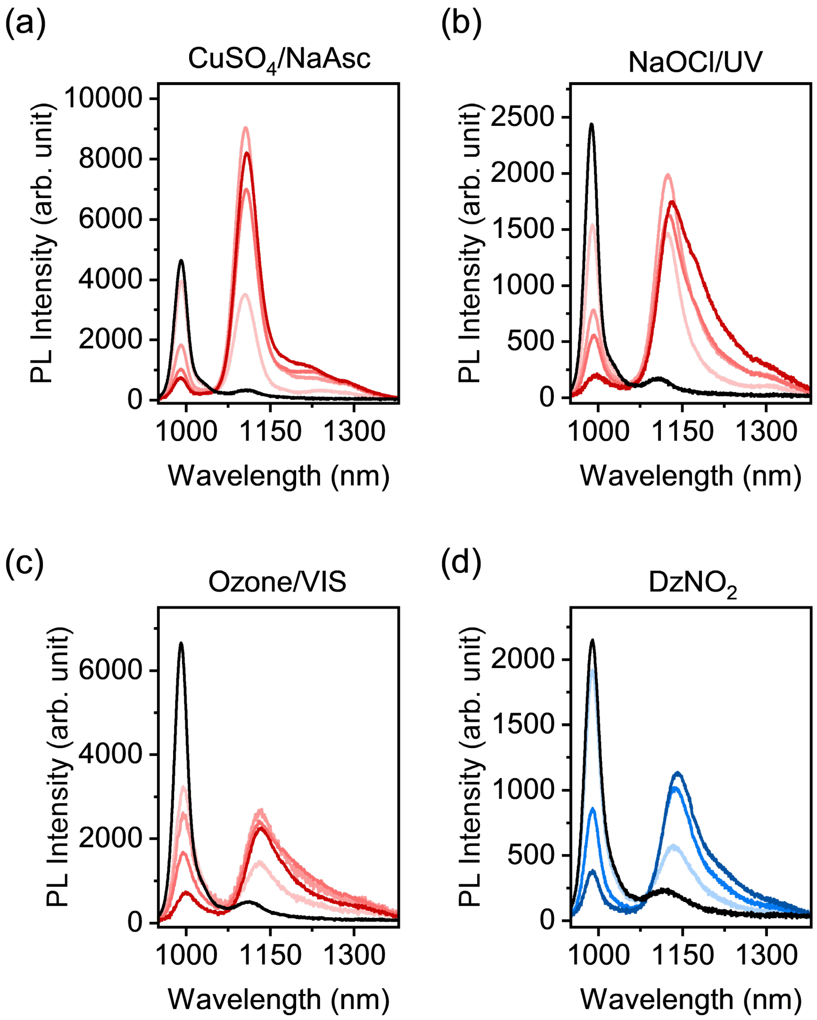

Figure S4. Absolute PL spectra of (6,5) SWCNTs functionalized with luminescent oxygen and aryl defects by different synthetic methods (**(a)** $CuSO_4$/NaAsc, **(b)** NaOCl/UV-light, **(c)** ozone/visible light, **(d)** $DzNO_2$).

E_{11}*/E_{11} PL intensity ratios for different functionalization methods

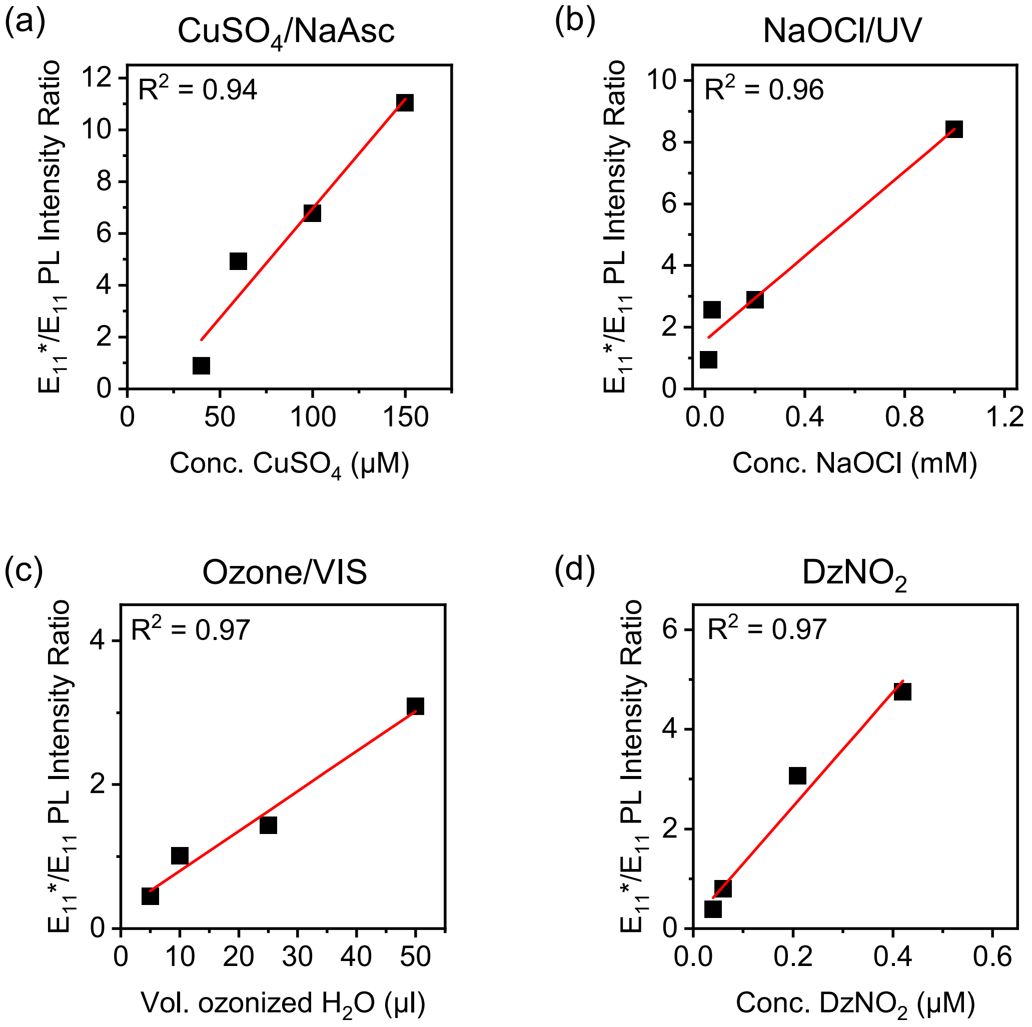

Figure S5. Dependence of the E_{11}*/E_{11} PL intensity ratios on the reactant concentration for (6,5) SWCNTs functionalized with luminescent oxygen and aryl defects by different synthetic methods (**(a)** $CuSO_4$/NaAsc, **(b)** NaOCl/UV-light, **(c)** ozone/visible light, **(d)** $DzNO_2$). Red lines are linear fits to the data (respective R^2 values are noted in the graphs).

Raman spectra of p-doped pristine and functionalized SWCNTs

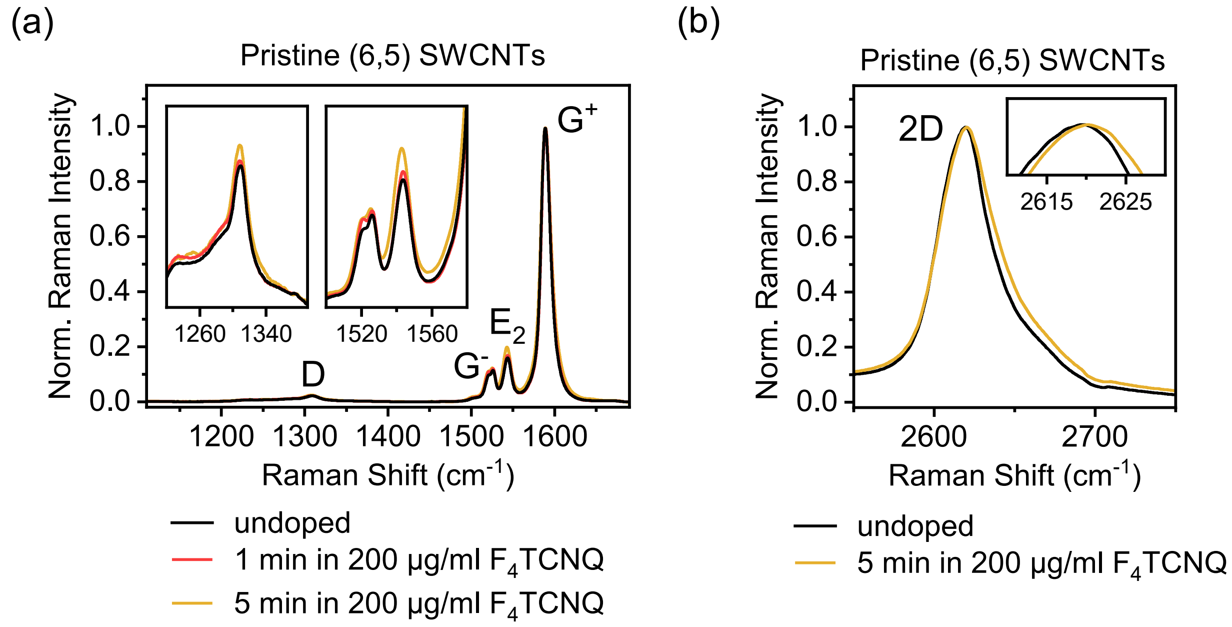

Figure S6. Influence of chemical doping on Raman spectra of (6,5) SWCNTs. **(a)** Averaged and normalized (to G^+-mode) Raman spectra of pristine (6,5) SWCNTs (drop cast film) without treatment and after 1 min and 5 min of immersion in a solution of the p-type dopant F_4TCNQ (solution in toluene, 200 µg/ml). **(b)** Averaged and normalized Raman spectra of the 2D-mode of pristine (6,5) SWCNTs before and after doping.

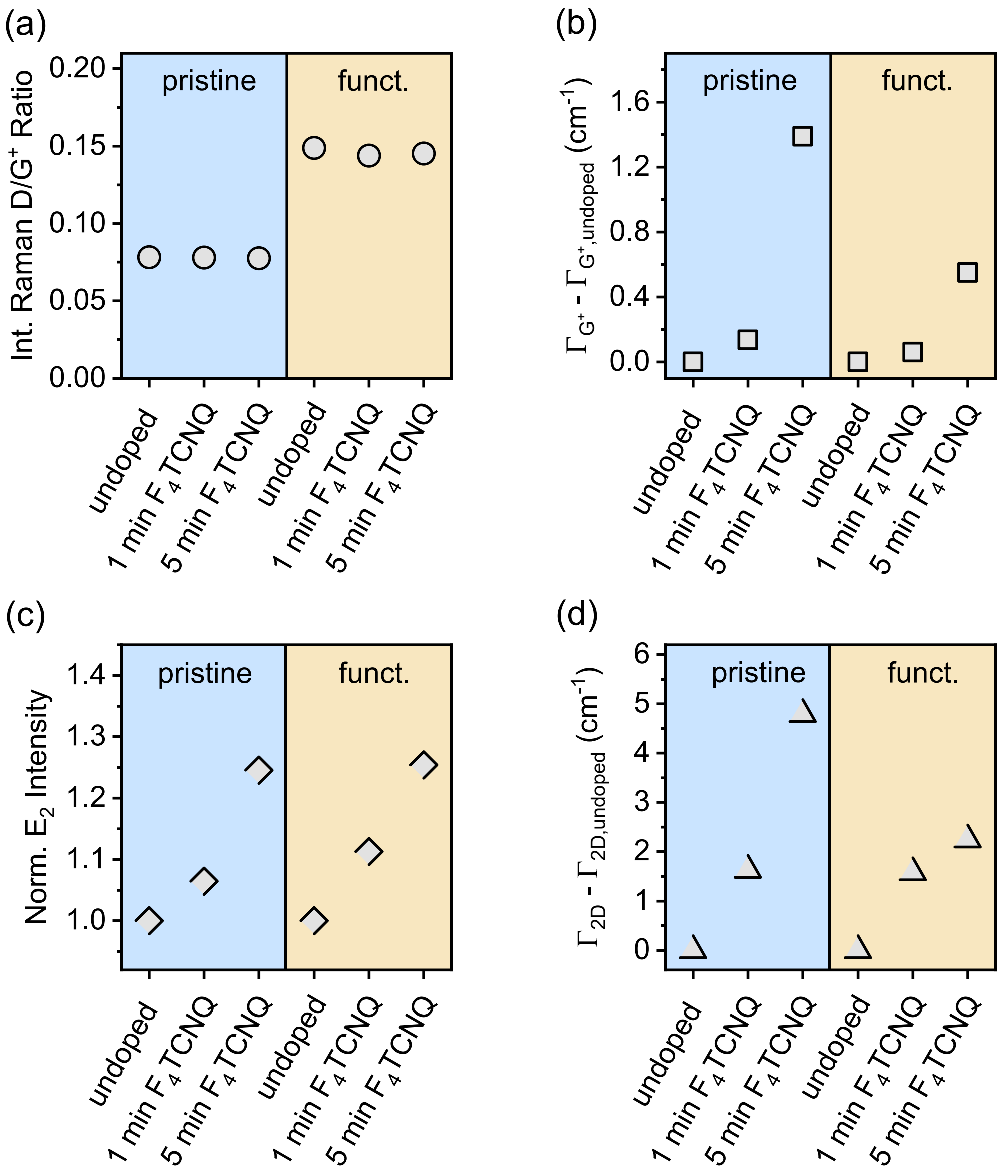

Figure S7. **(a)** Integrated Raman D/G^+ ratios for undoped/doped SWCNTs (pristine and functionalized with $CuSO_4$/NaAsc). **(b)** Raman G^+-mode linewidth (Γ_{D+}, full width at half maximum) for doped SWCNTs relative to the initial linewidth of the undoped sample. **(c)** Normalized intensity of the Raman E_2-mode for undoped/doped SWCNTs. **(d)** Linewidth of Raman 2D-mode (Γ_{2D}) for doped SWCNTs relative to the initial linewidth of the undoped sample.

Raman Δ(D/G$^+$) *vs* calculated defect density n_d for different functionalization methods

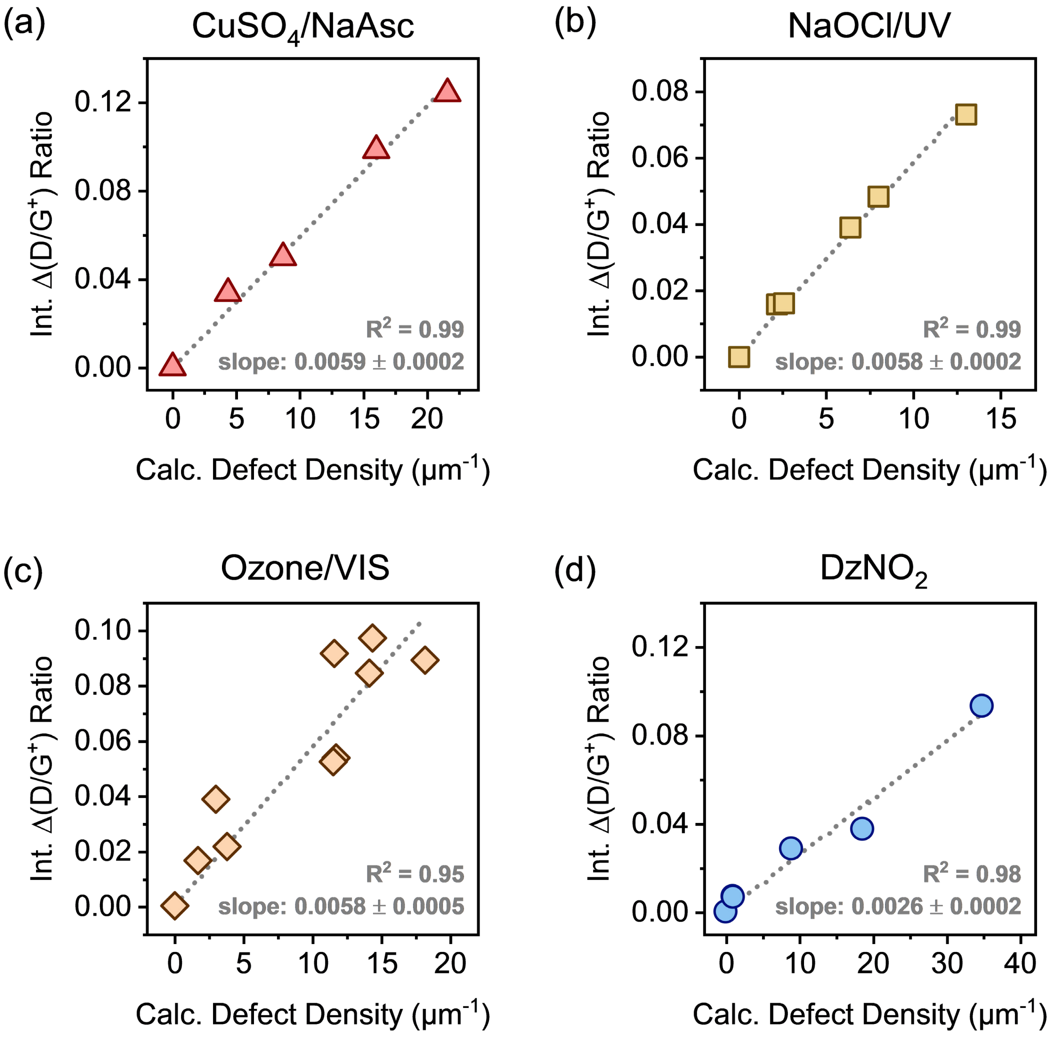

Figure S8. Integrated Raman Δ(D/G$^+$) ratios versus calculated defect density n_d (µm^{-1}) from PLQY measurements for (6,5) SWCNTs functionalized with luminescent oxygen and aryl defects via different synthetic methods (**(a)** $CuSO_4$/NaAsc, **(b)** NaOCl/UV-light, **(c)** ozone/visible light, **(d)** $DzNO_2$). Dashed grey lines are linear fits to the data (slope of the fit and respective R^2 values are noted in the graphs).

Raman spectroscopy of pristine and functionalized SWCNTs (RBM and IFM region)

The impact of luminescent oxygen and aryl defects on Raman modes other than the D- and G^+-mode should also be evaluated. In the spectral region between the radial breathing mode (RBM) and D-mode of SWCNTs, so-called intermediate frequency modes (IFMs) with weaker absolute signal intensities can be identified. For some IFMs dispersive characteristics in accordance with the involvement of double-resonant scattering processes were reported that are indicative of a defect-related origin.[12] Recently, we have demonstrated that the intensities of those IFMs, which can be derived from the ZA and ZO phonon branches in graphene, increase linearly with the defect density calculated from the E_{11} PLQY. This relation provides an additional method for the absolute quantification of luminescent defects in SWCNTs.[13] Furthermore, due to their chirality-dependent frequency, defect-related IFMs can also be applied for a relative quantification of the defect density in SWCNT mixtures.[14]

We recorded Raman spectra of drop-cast films of oxygen- and aryl-functionalized (6,5) SWCNTs in the IFM region using a 785 nm excitation laser. Averaged Raman spectra for all functionalization methods are shown in **Figure S9**. In all spectra the ZA-derived IFM can be identified at ~480 cm^{-1} whereas the ZO-derived IFM appears at ~580 cm^{-1}. For a quantitative evaluation the integrated IFM intensities were divided by the integrated intensity of the RBM of (6,5) SWCNTs (308 cm^{-1}) and the change of the integrated ratio, Δ(IFM/RBM), was correlated with the calculated defect density n_d. Irrespective of the functionalization method, a linear relation between Δ(IFM/RBM) and n_d was found although with significantly steeper slopes for oxygen defects compared to aryl defects, similar to the results reported for the D/G^+ ratios (Figure 3a). Individual slopes of Δ(IFM/RBM) *vs* n_d are presented in **Figure S10**. The low absolute Raman intensities of IFMs lead to some deviations of the Δ(IFM/RBM) *vs* n_d slopes for different methods of oxygen defect functionalization. However, the overall trend clearly supports the findings for the Raman $\Delta(D/G^+)$ ratio. For both the slope of Δ(ZA/RBM) and Δ(ZO/RBM) *vs* n_d, the values are larger by a factor of 1.9 for oxygen defects compared to aryl defects (see **Figure S11**).

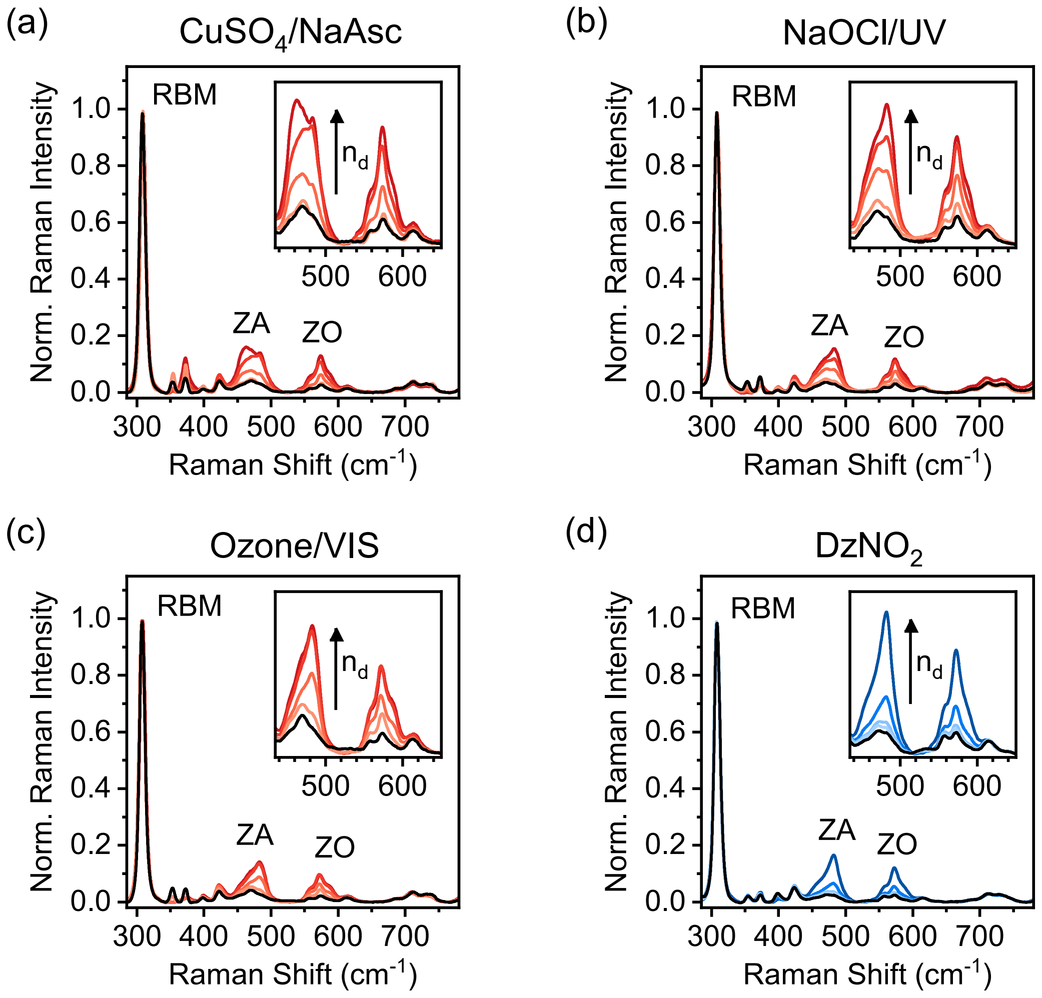

Figure S9. Averaged and normalized (to RBM at 308 cm^{-1}) Raman spectra of pristine and functionalized (6,5) SWCNTs for all functionalization methods; **(a)** $CuSO_4$/NaAsc, **(b)** NaOCl/UV-light, **(c)** ozone/visible light, **(d)** $DzNO_2$, λ_{exc} = 785 nm, >3600 individual spectra per sample. Insets display the evolution of the intermediate frequency modes (IFMs) for increasing degree of functionalization.

Raman Δ(IFM/RBM) *vs* calculated defect density n_d for different functionalization methods

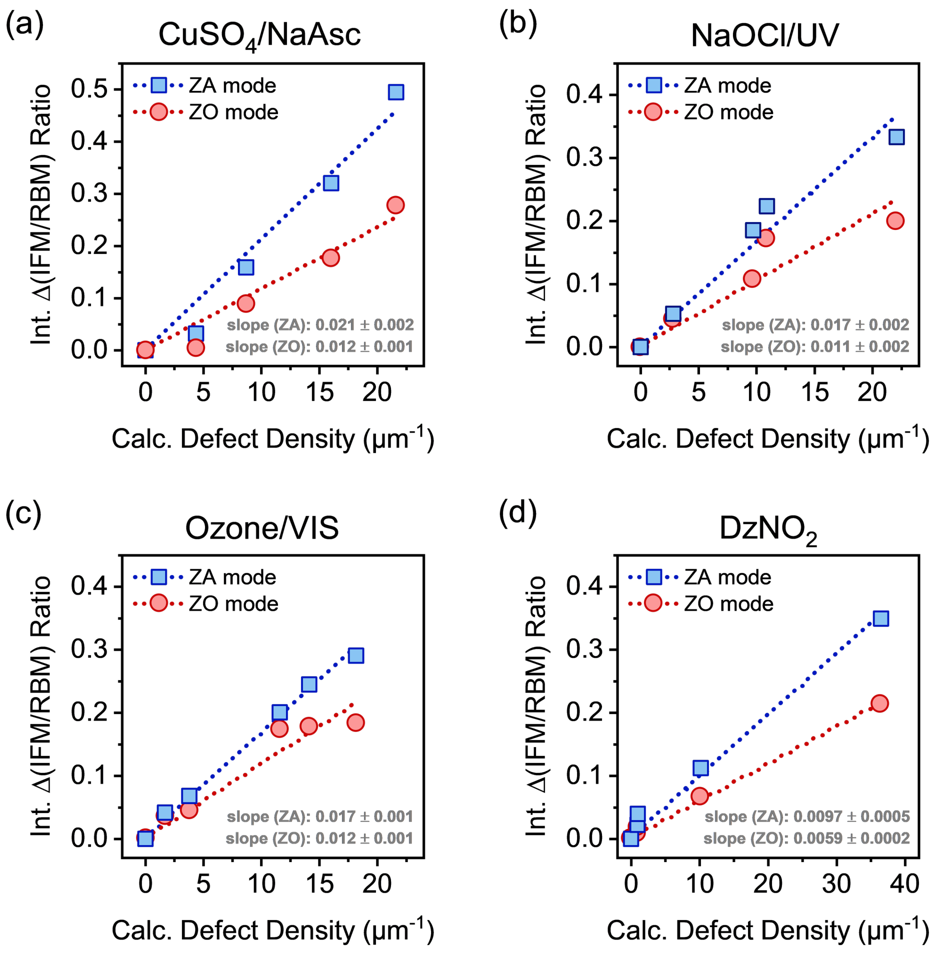

Figure S10. Integrated Raman Δ(ZA/RBM) and Δ(ZO/RBM) ratios versus calculated defect densities n_d for pristine and functionalized (6,5) SWCNTs (drop cast films) for all functionalization methods; **(a)** $CuSO_4$/NaAsc, **(b)** NaOCl/UV-light, **(c)** ozone/visible light, **(d)** $DzNO_2$. Dashed lines are linear fits to the data (slopes of the fits and respective R^2 values are noted in the graphs).

Extracted Raman Δ(IFM/RBM) *vs* n_d slopes

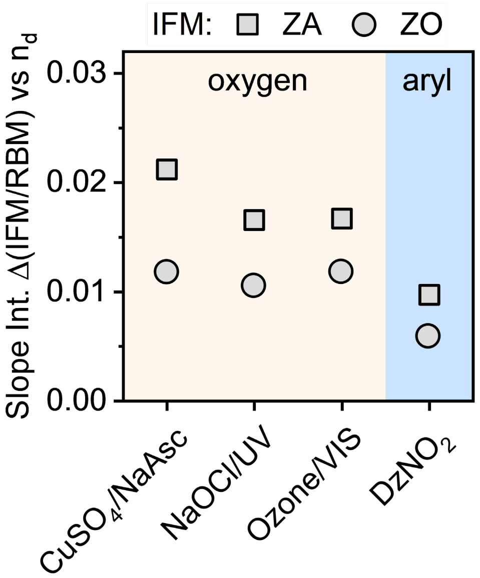

Figure S11. Extracted slopes of the integrated Δ(IFM/RBM) ratios versus calculated defect densities n_d for different functionalization methods.

Additional low-temperature single-SWCNT PL spectra

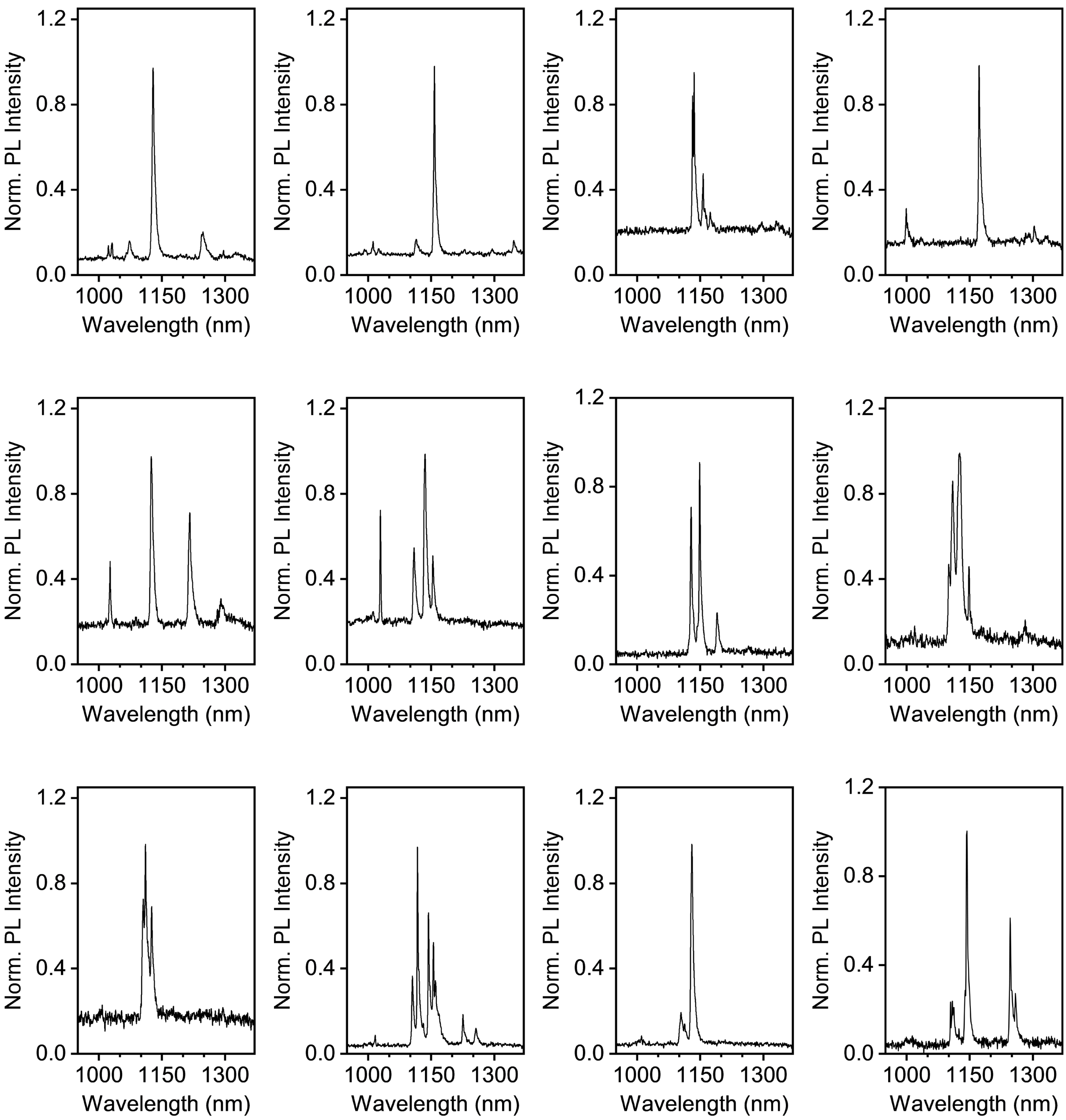

Figure S12. Additional low-temperature (4.7 K) PL spectra of single SWCNTs functionalized with $CuSO_4$/NaAsc, embedded in a polystyrene matrix (continued on the next page).

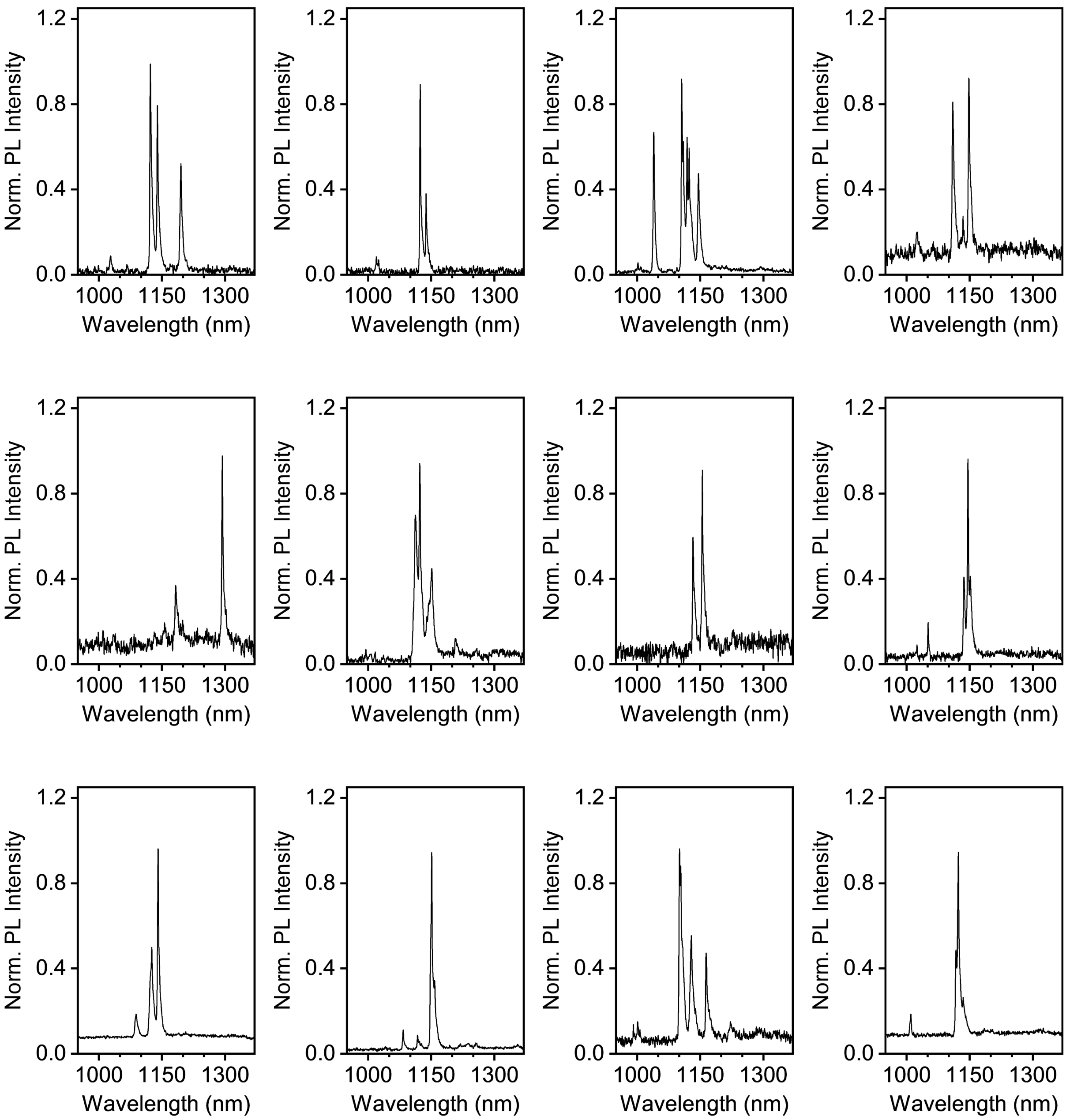

Figure S13. Additional low-temperature (4.7 K) PL spectra of single SWCNTs functionalized with $CuSO_4$/NaAsc, embedded in a polystyrene matrix.

AFM length statistics of functionalized SWCNTs

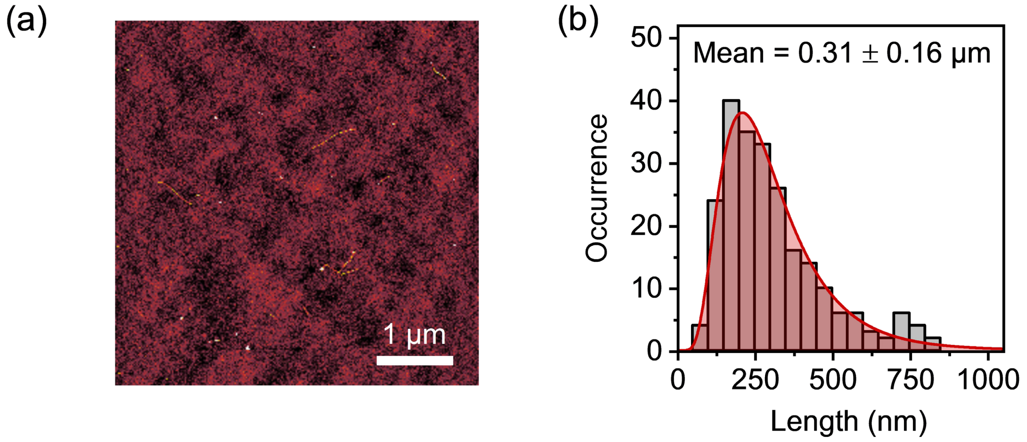

Figure S14. **(a)** Representative Atomic force micrograph and **(b)** length histogram (log-normal distribution, red line) of functionalized (6,5) SWCNTs ($CuSO_4$/NaAsc) after transfer from aqueous dispersion (0.3 % w/v SDS) to toluene/PFO-BPy.

References

(1) Sebastian, F. L., Zorn, N. F., Settele, S., Lindenthal, S., Berger, F. J., Bendel, C., Li, H., Flavel, B. S. and Zaumseil, J. Absolute Quantification of sp^3 Defects in Semiconducting Single-Wall Carbon Nanotubes by Raman Spectroscopy. *J. Phys. Chem. Lett.* **2022**, *13*, 3542-3548.

(2) Settele, S., Stammer, F., Sebastian, F. L., Lindenthal, S., Wald, S. R., Li, H., Flavel, B. S. and Zaumseil, J. Easy Access to Bright Oxygen Defects in Biocompatible Single-Walled Carbon Nanotubes Via a Fenton-Like Reaction. *ACS Nano* **2024**, *18*, 20667-20678.

(3) Lin, C.-W., Bachilo, S. M., Zheng, Y., Tsedev, U., Huang, S., Weisman, R. B. and Belcher, A. M. Creating Fluorescent Quantum Defects in Carbon Nanotubes Using Hypochlorite and Light. *Nat. Commun.* **2019**, *10*, 2874.

(4) Ghosh, S., Bachilo, S. M., Simonette, R. A., Beckingham, K. M. and Weisman, R. B. Oxygen Doping Modifies near-Infrared Band Gaps in Fluorescent Single-Walled Carbon Nanotubes. *Science* **2010**, *330*, 1656-1659.

(5) Pfohl, M., Tune, D. D., Graf, A., Zaumseil, J., Krupke, R. and Flavel, B. S. Fitting Single-Walled Carbon Nanotube Optical Spectra. *ACS Omega* **2017**, *2*, 1163-1171.

(6) Graf, A., Zakharko, Y., Schießl, S. P., Backes, C., Pfohl, M., Flavel, B. S. and Zaumseil, J. Large Scale, Selective Dispersion of Long Single-Walled Carbon Nanotubes with High Photoluminescence Quantum Yield by Shear Force Mixing. *Carbon* **2016**, *105*, 593-599.

(7) Berger, F. J., Lüttgens, J., Nowack, T., Kutsch, T., Lindenthal, S., Kistner, L., Müller, C. C., Bongartz, L. M., Lumsargis, V. A., Zakharko, Y. and Zaumseil, J. Brightening of Long, Polymer-Wrapped Carbon Nanotubes by sp^3 Functionalization in Organic Solvents. *ACS Nano* **2019**, *13*, 9259-9269.

(8) Hertel, T., Himmelein, S., Ackermann, T., Stich, D. and Crochet, J. Diffusion Limited Photoluminescence Quantum Yields in 1-D Semiconductors: Single-Wall Carbon Nanotubes. *ACS Nano* **2010**, *4*, 7161-7168.

(9) Hofmann, M. S., Glückert, J. T., Noe, J., Bourjau, C., Dehmel, R. and Högele, A. Bright, Long-Lived and Coherent Excitons in Carbon Nanotube Quantum Dots. *Nat. Nanotechnol.* **2013**, *8*, 502-505.

(10) Raynaud, C., Claude, T., Borel, A., Amara, M. R., Graf, A., Zaumseil, J., Lauret, J.-S., Chassagneux, Y. and Voisin, C. Superlocalization of Excitons in Carbon Nanotubes at Cryogenic Temperature. *Nano Lett.* **2019**, *19*, 7210-7216.

(11) Settele, S., Berger, F. J., Lindenthal, S., Zhao, S., El Yumin, A. A., Zorn, N. F., Asyuda, A., Zharnikov, M., Högele, A. and Zaumseil, J. Synthetic Control over the Binding Configuration of Luminescent sp^3-Defects in Single-Walled Carbon Nanotubes. *Nat. Commun.* **2021**, *12*, 2119.

(12) Fantini, C., Jorio, A., Souza, M., Ladeira, L. O., Souza Filho, A. G., Saito, R., Samsonidze, G. G., Dresselhaus, G., Dresselhaus, M. S. and Pimenta, M. A. One-Dimensional Character of Combination Modes in the Resonance Raman Scattering of Carbon Nanotubes. *Phys. Rev. Lett.* **2004**, *93*, 087401.

(13) Sebastian, F. L., Becker, F., Yomogida, Y., Hosokawa, Y., Settele, S., Lindenthal, S., Yanagi, K. and Zaumseil, J. Unified Quantification of Quantum Defects in Small-Diameter Single-Walled Carbon Nanotubes by Raman Spectroscopy. *ACS Nano* **2023**, *17*, 21771-21781.

(14) Soltani, N., Zheng, Y., Bachilo, S. M. and Weisman, R. B. Structure-Resolved Monitoring of Single-Wall Carbon Nanotube Functionalization from Raman Intermediate Frequency Modes. *J. Phys. Chem. Lett.* **2023**, *14*, 7960-7966.
